# Easy flow: An eclectic cardiopulmonary bypass simulation model

**DOI:** 10.1177/02676591241261017

**Published:** 2024-06-10

**Authors:** Ishaq I Najmuddin, P Sainath, PR Srinidhi

**Affiliations:** Perfusion Technology, 76799 Manipal College of Health Professions, Manipal, India

**Keywords:** cardiopulmonary bypass, simulation, perfusion education, extracorporeal technology, healthcare

## Abstract

The aphorism “Primum non nocere” underscores the responsibility of healthcare professionals to prioritize patient safety. Perfusionists, experts in extracorporeal circulation, play a pivotal role in maintaining cardiac function during critical situations. However, the evolving landscape of medical technology has not been without challenges, particularly in ensuring equitable access to perfusion training. Easy Flow Cardiopulmonary Bypass Simulation Model stands out as a cost-effective alternative, utilizing routine CPB equipment found in operating theatres. The uniqueness lies in its incorporation of double reservoirs and two pumps, a novel approach not reported before in educational CPB simulation models. The benefits of this model extend beyond skill development to encompass team management, communication enhancement, and disaster management training. Multiple scenarios, from the initiation of CPB to addressing emergencies like massive air embolism, can be simulated. Although the model requires an instructor, this facilitates the integration of essential professional and communication skills into training. The adaptability of the Easy Flow model makes it a practical and sustainable solution. It provides hands-on experience for perfusion students, translating theoretical knowledge into practical competence. The model's simplicity, combined with its use of readily available materials, positions it to be an accessible tool for educational institutions and healthcare centers globally. In conclusion, the Easy Flow CPB Simulation Model not only fills a critical gap in perfusion education but also exemplifies how innovation can bridge disparities, ensuring that quality healthcare education is within reach for all. Its potential impact on global healthcare training is profound, promising a future where knowledge sharing leads to improved patient care.

## Introduction

“Primum non nocere,” a phrase that translates to “First, do no harm,” is an essential part of the medical oath and the daily lives of healthcare professionals in the modern era.

One such camouflaged healthcare professional is a perfusionist who is an expert at establishing, monitoring and maintaining extracorporeal circulation using various technologies.^1^ Perfusionists are highly skilled healthcare professionals who play a vital role in complex cardiovascular surgeries. Their expertise lies in operating and managing the heart-lung machine, essentially taking over the heart’s function during critical interventions.

The world of medicine thrives on innovation, with advanced technology constantly pushing the boundaries of patient care. Yet, amidst these advancements lies a stark reality; equitable access to resources remains a critical challenge. When it comes to perfusion training, high-fidelity simulators often come with hefty price tags, leaving them out of reach for many healthcare institutions. This creates a gap in crucial skill development, potentially impacting patient outcomes in circumstances where it matters most.

Cardiopulmonary bypass (CPB), an extracorporeal method frequently used in open-heart surgery, is used to temporarily assist the body’s respiratory and cardiac functions. Cardiopulmonary bypass is a life-saving technique, but because of human errors or device malfunctions, it can cause serious harm or even death to a patient.^2^ Hence perfusionists require well-rounded training in standard and emergency procedures. Simulator-based education and evaluation methods could aid in their development while promoting the creation of more advanced medical simulators. This comprehensive training equips perfusionists to handle any situation.^3^

For a perfusionist, it is important to ensure carefulness in their practice to avoid major and minor accidents. To minimize the occurrence of errors, educational institutes have tried to incorporate simulation labs for perfusion education which has proven to improve a student’s hand-to-eye coordination and problem-solving skills which are both of utmost importance when it comes to unfortunate events. The importance of experience in simulators is that, prior to the start of clinical practice, students gain basic skills related to CPB and develop their ability to deal with various hemodynamic conditions.^4^

The impact of simulation extends beyond individual skill development. It cultivates a culture of knowledge-sharing and self-sufficiency. As more individuals gain access to quality training, the ripple effect is undeniable in terms of improved patient care and strengthened healthcare systems.

## Description

Here, we describe a new CPB simulation model we have innovated. It is a simple, yet effective simulation model solely based on the routine CPB equipment used in the operating theatre.

We have used two hard-shell reservoirs in our model, one of which is the standard venous reservoir, and the other is the pseudo-patient reservoir which holds the patient volume. Many simulation models have been reported where they have used two reservoirs, but these models were specifically used for research and have not been reportedly implemented in perfusion simulation education.^5–7^

The unique feature of our model is the inclusion of two roller pumps. One of which (patient pump) is used by the instructor to control and manage the venous drainage during the simulation scenarios, while the other is the conventional arterial pump in the CPB circuit. Our standard CPB circuit includes two recirculation lines, one for the oxygenator and one more in the arterial line to eliminate air between the arterial line and the oxygenator. The cardioplegia set-up in our circuit is mainly for blood-based cardioplegia where a ratio of blood to crystalloid is used. We have also added a suction pump in the pseudo-patient circuit which is connected to the venous line via a Y-connector to achieve venous airlock when the patient pump is off. Manipulation of the patient pump flows by an instructor provides scenarios such as low venous drainage and chattering of the venous line. Also, running the suction pump in reverse produces air entrainment in the venous line.

The equipment we have used is Terumo Sarns9000 Heart-Lung machine. The disposables we have used for the model are Maquet Quadrox-i Adult as the pseudo-patient reservoir, the Nipro-Vital as the venous reservoir and oxygenator, medically graded PVC tubing, and the Spictra MPS- Adult as the cardioplegia delivery device. We acquire our disposables from our affiliated hospital. They are vigorously washed with the help of the heart-lung machine which creates a positive pressure. The roller pump is used to water wash, followed by a thorough soap wash, after which it is water washed twice or thrice to remove the soap residue. It is ensured that any visible blood components are eliminated and the disposables are disinfected. When acquiring the disposables, CPB circuits that have been used for cancer patients and for patients with infections that can be transmitted through blood, are excluded. These steps help minimize the risk of infections for both students and tutors. Also, expired products are acquired when available.

To display patient vital signs on a monitor and manipulate it according to the alterations in the flow and scenarios, we equipped an application called Simpl – A simulated patient monitor (developed by SimulationSense) and displayed it on a screen. The controller device was handled by the instructor. Other hemostatic monitoring such as arterial blood gas (ABG) analysis and activated clotting time (ACT) values are verbally communicated or a printed sheet of the values is given to the trainees performing the simulation, by the instructor. Assembling the model circuit takes approximately 15 min and priming and initiating simulation may require additional time based on the level of skill. In our simulation model, we incorporate a screen that conceals the operator intervention from the student. The screen is placed in such a way that the student cannot view the pseudo pump and the suction pump while also concealing the pseudo-patient reservoir.

This simulation model requires an instructor to be present near the pseudo-patient circuit to control the pumps. Students are briefed on the conduct of the simulation during their first few simulation sessions, where the instructor demonstrates how the simulator works. Following the completion of pre-bypass protocols, simulation begins with initiation of CPB. The instructor will give the commands “Go on bypass”, the arterial pump is run, and the venous line is unclamped by the student. Simultaneously, the instructor must slowly start running the patient pump to regulate the inflow of the venous reservoir volume. The instructor observes the student who performs the simulation and provides verbal feedback during and after the session.

Our model can be easily modified according to institutional guidelines, Figurs 1 and 2 such as addition of an arterial filter, bubble trap, hemoconcentrator and incorporation of cytosorb if available. Safety devices such as level sensor and bubble detector may also be used.

**Figure 1.**
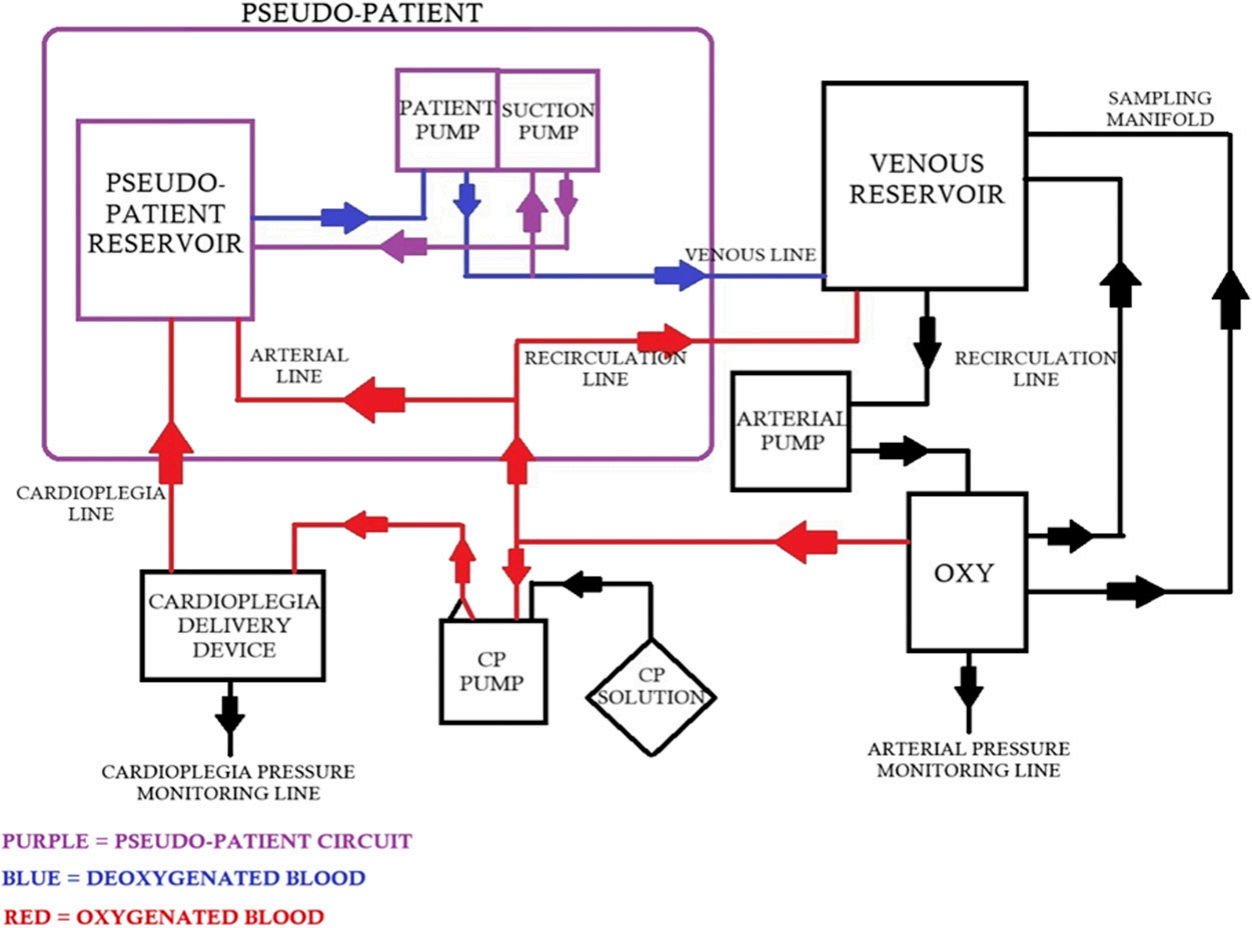
Schematic circuit diagram of the Easy Flow cardiopulmonary bypass simulation model.

**Figure 2.**
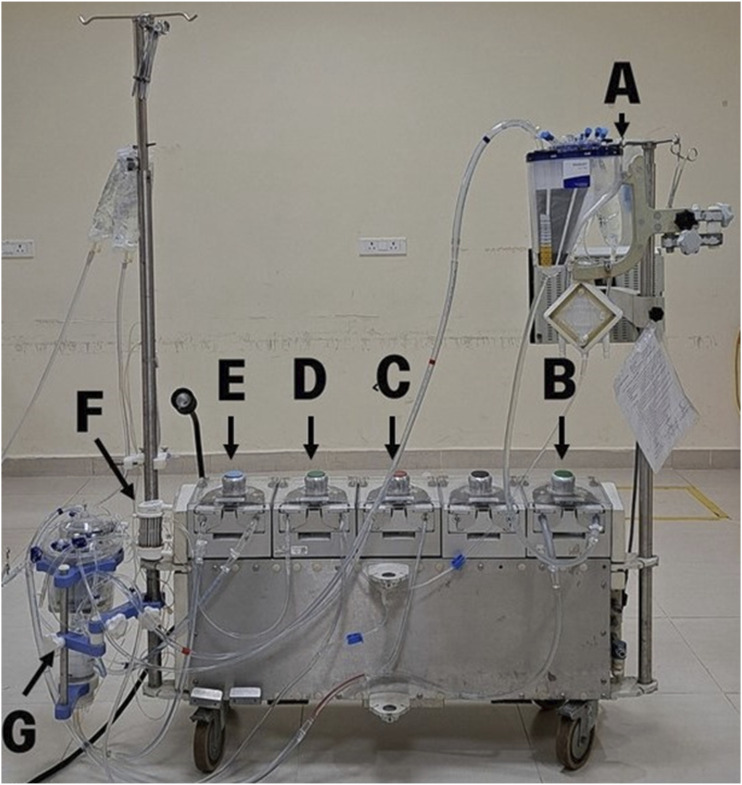
Representative image of Easy Flow cardiopulmonary bypass simulation model; A- Pseudo-patient reservoir, B- Pseudo-patient pump, C- Suction pump, D- cardioplegia pump, E- Arterial pump, F-Cardioplegia delivery device, G- Venous reservoir and Oxygenator (Nipro-Vital).

## Discussion

The incorporation of double reservoirs and double pumps in an educational CPB simulation model has not been reported before. We take pride in providing a cost-effective and easy-to-assemble model to enhance perfusion education alongside hands-on practice.

The model has many possible objectives like skill training, communication, professional behavior enhancement, establishing new team management protocols, evaluating students’ capabilities, evaluating functioning of new or existing perfusion equipment or techniques, research and development, advancement in non-technical skills such as decision making and clinical disaster management.^8–10^

Multiple scenarios can be portrayed and practiced using this model, such as assembling and priming of the CPB circuit, practice of pre-bypass protocol with a checklist, steps of initiation and termination of CPB, management of low venous drainage, venous airlock, venous air entrainment, administration of cardioplegia doses, action required when aortic cross clamp is placed or removed and conduct of different types of ultrafiltration. Several accidents such as electrical failure, oxygenator change-out in case of oxygenator failure, pump-head rupture, pump failure and massive air embolism can also be shown, and the management may be practiced.

Our model requires an instructor to be present, which can be counted as a disadvantage compared to the automated simulators available these days. Although the presence of an instructor can be an additional task, it allows us to incorporate important agendas to the simulation training such as enhancement of professional behavior, communication skills and training on inter-professional skills.^11^

We have envisioned a perfusion simulation model that prioritizes practicality and utilization of readily available materials. This is a new avenue for learners in underprivileged regions, empowering them with the tools they need to hone their skills and equip them in providing quality care, as perfection comes with practice. Perfusion students gain invaluable hands-on experience, solidifying theoretical knowledge into practical application. This translates to greater confidence and competence when facing real operation theatre scenarios. Resource-limited hospitals, especially in countries like India can train their perfusionists effectively without straining their budgets.

The model on its own is quite adaptable, whereby scenarios can be created to address specific needs and clinical practices, by modification of the circuit, ensuring its relevance and effectiveness. Easy maintenance and readily available materials ensure that this model has a long-term use and minimizes dependence on external resources, proving its sustainability.

## Conclusion

Power lies in sharing knowledge. We believe since the equipment that is required to utilize this simulation model is not difficult to obtain, with regards to availability and cost effectiveness, educational institutions and healthcare centers that are looking for better simulation models to incorporate can use this model as a quick and easy solution. In a world often divided by disparities, this stands as an example of how ingenuity can bridge the gap and pave the way for a future where quality healthcare is truly accessible to all.
